# β-Aminopropioamidoximes derivatives as potential antitubercular agents against anthropozoonotic infections caused by *Mycobacterium tuberculosis* and *Mycobacterium bovis*

**DOI:** 10.14202/vetworld.2025.731-745

**Published:** 2025-03-31

**Authors:** Lyudmila Kayukova, Venera Bismilda, Kairat Turgenbayev, Assem Uzakova, Gulnur Baitursynova, Umirzak Jussipbekov, Meruyert Mukanova, Lyailya Chingissova, Gulnur Dyussembayeva, Assiya Borsynbayeva, Azamat Yerlanuly, Ablay Auyezov

**Affiliations:** 1 JSC A.B. Bekturov Institute of Chemical Sciences, Laboratory of Chemistry of Synthetic and Natural Drug Substances, Almaty, Kazakhstan; 2 National Scientific Center of Phthisiopulmonology, Ministry of Health of the Republic of Kazakhstan, National Reference Bacteriological Laboratory, Almaty, Kazakhstan; 3 LLP Scientific and Production Center BioVet, Almaty, Kazakhstan

**Keywords:** antitubercular agents, *Mycobacterium bovis*, *Mycobacterium tuberculosis*, SwissADME, tuberculosis, β-aminopropioamidoximes

## Abstract

**Background and Aim:**

Tuberculosis (TB) remains a significant global health challenge, with increasing incidences of drug-sensitive (DS) and multidrug-resistant (MDR) TB. In addition, *Mycobacterium bovis*-induced zoonotic TB (zTB) presents treatment difficulties due to its resistance to pyrazinamide and the prolonged treatment duration required. This study aims to evaluate the antitubercular potential of β-aminopropioamidoxime derivatives against DS and MDR *M. tuberculosis* and *M. bovis* strains, and utilizing the SwissADME prognostic tool to predict the drug- and lead-likeness of the described compounds.

**Materials and Methods:**

Six β-aminopropioamidoxime derivatives were synthesized through O-aroylation of amidoxime followed by dehydration to form 1,2,4-oxadiazoles. The compounds were tested *in vitro* against DS, MDR *M. tuberculosis*, and *M. bovis* using Sotton’s liquid medium and subcultured on dense Lowenstein-Jensen medium. SwissADME was used to predict drug-likeness and pharmacokinetic properties.

**Results:**

The derivatives exhibited significant antitubercular activity, with *in vitro* efficacy 5–100 times greater than rifampicin. 1,2,4-oxadiazoles with *para*-bromo and *meta*-chloro substituents demonstrated the highest activity against DS and MDR *M. tuberculosis*, while O-*para-*toluoyl-β-(morpholin-1-yl)propioamidoxime salts (hydrochloride, oxalate and citrate) were 10 times more active against *M. bovis*. SwissADME analysis confirmed favorable pharmacokinetic properties, including high gastrointestinal absorption and drug-likeness, with lead-likeness identified in four compounds.

**Conclusion:**

The study presents β-aminopropioamidoxime derivatives as promising candidates for antitubercular therapy against both human and zTB. Their enhanced activity, oral bioavailability, and potential integration into new treatment regimens underscore their therapeutic relevance. Further *in vivo* studies are recommended to validate their efficacy and safety for clinical applications.

## INTRODUCTION

In recent years, there has been an increase in the global incidence of tuberculosis (TB) associated with several factors related to TB treatment during the COVID-19 pandemic [1, 2]. The emergence of *Mycobacterium tuberculosis* resistance has been attributed to the prolonged use of standardized regimens containing four first-line drugs, namely rifampicin, isoniazid, pyrazinamide, and ethambutol or streptomycin, for 6–9 months in treating drug-susceptible TB [3]. In addition, the treatment of drug-resistant (DR), multidrug-resistant (MDR) TB is hindered by low drug efficacy, toxicity associated with treatment, and high costs, requiring a treatment duration of 20–24 months, primarily involving fluoroquinolones and second-line injectables, such as amikacin, capreomycin, or kanamycin [4–6]. The costs associated with MDR-TB treatment range from $650 to $8,266/patient. The costs associated with treating MDR-TB patients are significantly higher than those for DS-TB, costing patients a 20% of their annual household income [7].

Since the 1990s, several attempts have been made to determine treatment regimens that can overcome the shortcomings of existing regimens, including both new and repurposed drugs, in response to the urgent need for more effective treatment regimens for all types of DR TB. As of August 2023, 28 drugs for the treatment of TB have entered Phases I, II, and III trials [8].

The key change in the latest World Health Organization (WHO) recommendations is the addition and prioritization of a new all-oral 6-month (BPaLM) regimen for the treatment of DR TB [1].

TB is defined by 12 closely related members of the *Mycobacterium* *genus*, termed the *M. tuberculosis* complex, with *M. tuberculosis* and *M. bovis*, among others [9-12]. *M. bovis* exhibits almost complete resistance to pyrazinamide. Therefore, antibiotic regimens based on first-line anti-tubercular drugs (rifampicin, isoniazid, and ethambutol) should be considered. In addition, the duration of treatment for *M. bovis* is longer than that for *M. tuberculosis* (9 vs. 6 months) [13].

Of the 10 million newly reported cases of TB, with 1.6 million deaths, approximately 142,000 new cases and 12,500 deaths were attributed to *M. bovis* [14]. Termed “zoonotic TB,” *M. bovis* cases in humans are likely underestimated due to a lack of reporting in endemic countries and limited laboratory capacity [15-17]. TB caused by *M. bovis* is well-defined by the World Organization for Animal Health, which comprises 179 countries [18].

The transmission routes of *M. bovis* remain poorly understood. It is generally assumed that transmission occurs through animals, mainly by aerosols. Humans may also transmit disease through the consumption of contaminated animal products such as raw milk, dairy products, and meat. In addition, contact with infected body fluids or tissues is another route of transmission that has a higher probability for slaughterhouse workers and hunters. In countries with TB surveillance programs, infected livestock is usually removed before developing clinical signs [19]. The impact of *M. bovis* epidemiology is growing, given the serious economic consequences: in international trade due to import bans on infected animals and animal products; in wildlife, species are driven to extinction; and in businesses, such as tourism, the impact is difficult to assess [20].

The misuse of anti-TB drugs leads to resistance in animals, raising global concerns regarding the potential transmission of the pathogen to humans [21].

Research under the framework of the “One Health” concept using computational methods can identify and type bacteria and propose new strategies to combat MDR bacteria in human and veterinary medicine. Sharing resources and increasing collaboration between public health and veterinary scientists will increase awareness of the “shared risk” of TB in humans and animals and, in resource-limited situations, maximize the use of existing infrastructure and reduce unnecessary duplication of efforts in disease control programs [22-24].

The new 6-month DR TB regimen (BPaLM or BPaLC) recommended by the WHO was based on the results of the TB-PRACTECAL trial, which was completed in 2022. The 6-month regimen in MDR-TB patients consists of the following new or repurposed drugs: bedaquiline, pretomanid, linezolid, and moxifloxacin (or clofazimine) [25].

**Figure 1:**
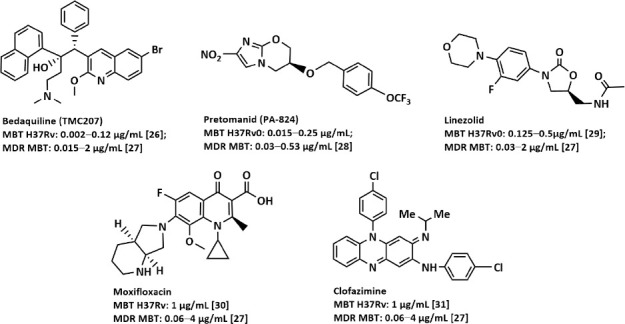
Preparations included in the shortened 6-month treatment regimen for patients with MDR/RR-TB or MDR/RR-TB with additional resistance to fluoroquinolones (pre-XDR-TB). MDR=Multidrug-resistant, RR-TB=Rifampicin-resistant tuberculosis, XDR-TB=Extensively drug-resistant tuberculosis [26–31].

Figure 1 illustrates the chemical structures of the drugs included in these regimens and their minimum bactericidal concentrations (MBCs) in the DS and MDR strains: bedaquiline [26, 27], pretomanid [28], linezolid [27, 29], moxifloxacin [27, 30], and clofazimine [27, 31].

In the early stages of drug development, the *in vitro* activity of pharmaceuticals and their chemical form are key factors. The bioavailability of possible dosage forms, water solubility, and stability are important. There is a risk of transformations during the non-clinical and clinical stages [32]. As a rule, the commercial appearance of the drugs that make up the TB-PRACTECAL regimen is in the form of salts: bedaquiline [33], linezolid [34], moxifloxacin [35], or as a suspension in an oil-wax base–clofazimine [36], or either in 0.5% methylcellulose or in cyclodextrin/lecithin (CM2)–pretomanid [37].

Long-term treatment for TB results in side effects, the most common of which are as follows: acute or chronic inflammation of the tendons, hearing and vision loss, liver and kidney damage, neuropathy, and QT prolongation [38]. The rapid emergence of bacteria resistant to existing treatments necessitates the development of new molecules with anti-tubercular properties and optimization of existing anti-TB drugs [39].

Due to the relevance of TB, especially DR forms, in Kazakhstan [40] and the difficulties in treating MDR and XDR TB, our research group has been actively searching for novel anti-tubercular drugs.

**Scheme 1:**
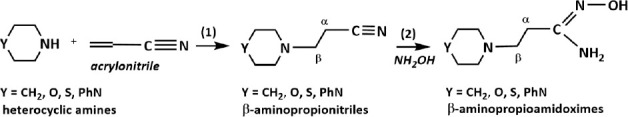
General method for the preparation of β-aminopropioamidoximes.

The key compounds selected for synthetic modifications are β-aminopropioamidoximes. Six-membered nitrogen-containing heterocycles with possible additional heteroatoms are used as β-amino substituents. The synthesis scheme consists of two stages: (1) the reaction of heterocyclic amines with acrylonitrile to form β-aminopropionitriles and (2) the production of β-aminopropioamidoximes in the reaction of β-aminopropionitriles with hydroxylamine (Scheme 1).

In the aroylation reactions of β-amino-propioamidoximes, only derivatives at the oxygen atom of the oxime fragment were obtained. The dehydration of O-aroyl derivatives leads to the formation of 3,5-disubstituted 1,2,4-oxadiazoles. The β-amino atom facilitates the generation of water-soluble salts. This is especially important in the development of anti-tubercular drugs, considering the duration of both DS and MDR TB treatment and the need to operate with a convenient water-soluble dosage form. In addition, the synthesis of the β-aminopropioamidoxime derivatives libraries was conducted based on the 3-4 stage scheme with good yields.

**Figure 2:**
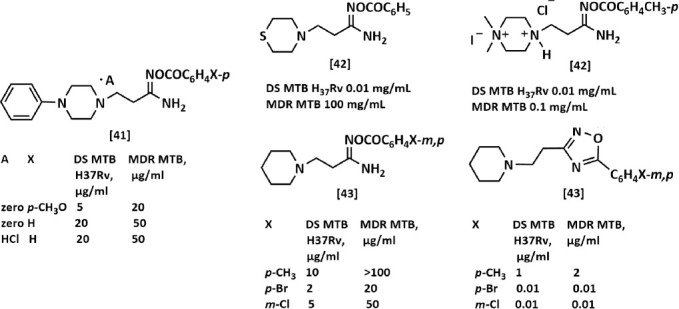
The most *in vitro* active samples on DS *M. tuberculosi*s H37Rv and MDR *M. tuberculosis* from the series of arylation products β-(4-phenylpiperazin-1-yl)- [41], β-(thiomorpholin-1-yl)- [42], β-(4-methylpiperazin-1-yl)- [42], β-(piperidin-1-yl)- [43] propioamidoximes and 1,2,4-oxadiazoles [43]. MTB=*M. tuberculosis*, DS=Drug sensitive, MDR=Multidrug-resistant.

Figure 2 shows the most *in vitro* active molecules against DS *M. tuberculosis* H37Rv and MDR *M. tuberculosis* O-aroyl-β-aminopropioamidoximes and 3,5-disubstituted 1,2,4-oxadiazoles obtained before this work [41–43].

In accordance with the “One Health” idea [22-24], we conducted parallel *in vitro* screening of a group of β-aminopropioamidoximes derivatives on DS and MDR strains of *M. tuberculosis* and on *M. bovis* strains

The significance of this work is the development of a library of compounds from the β-aminopropioamid-oxime class as potential antitubercular agents against anthropozoonotic infections caused by *M. tuberculosis* and *M. bovis*. Comparison of minimum inhibitory concentrations (MICs) of the β-aminopropioamidoxime derivatives we obtained with MICs of compounds from the different classes (bedaquiline – diarylquinoline; pretomanid – nitroimidazole; linezolid – oxazolidinone; moxifloxacin – fluoroquinolone; clofazimine – phenazine), which are components of the TB-PRACTECAL regimen for the treatment of MDR-TB, allows us to consider them competitive and have optimism for their further development. In the literature, there are no examples of systematic studies of new drugs within the One Health concept and in the area of β-aminopropioamidoximes as well.

The aim of this work was to demonstrate the potential of the β-aminopropioamidoxime derivatives developed by us in the treatment of human TB caused by DS and MDR *M. tuberculosis* and anthropozoonotic TB caused by *M. bovis*; in addition, based on available *in vitro* data, to determine their competitiveness with the components of the abbreviated BPaLM(C) regimen. Using the SwissADME prognostic product based on physicochemical data to evaluate drug- and lead-likeness of the group of compounds presented in the article.

**Scheme 2:**
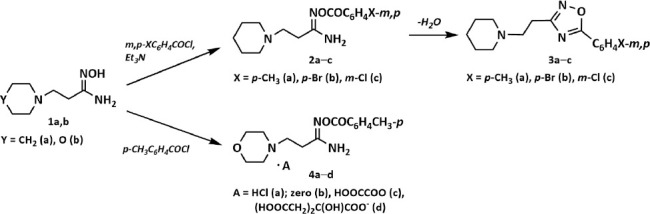
Synthesis of 5-aryl-3-[β-(piperidin-1-yl)ethyl]-1,2,4-oxadiazoles (**3a**-**c**) and derivatives of O-*para*-toluoyl-β-(morpholin-1-yl)propioamidoxime: hydrochloride (**4a**), base form (**4b**), oxalate (**4c**), citrate (**4d**).

Previously, we obtained 1,2,4-oxadiazoles (**3a**–**c**) derived from β-(piperidin-1-yl)propioamdoxime (**1a**) and studied their anti-TB properties on DS and MDR *M. tuberculosis* strains [43, 44]. Here, for the 1^st^ time, we present the results of *in vitro M. bovis* testing. For the 1^st^ time, we present the synthesis of a number of salts **4a, c, d** based on β-(morpholin-1-yl)propioamdoxime (**1b**) and the results of their testing on DS and MDR *M. tuberculosis* strains and on the *M. bovis* strain (Scheme 2 and Tables 1–4).

**Table 1:** Physicochemical data of O-*para*-toluoyl-β-(morpholin-1-yl) propioamidoxime (**4b**) and its pharmacologically acceptable salts: hydrochloride (**4a**), oxalate (**4c**), and citrate (**4d**).

Compound	Chemical form	Yield, %	mp, °C	*R*_f_	Gross formula	Solubility in water
**4a**	Hydrochloride	83.2	146	0.43	C_15_H_22_ClN_3_O_3_	Highly soluble
**4b**	Base	93.0	82‒6	0.49	C_15_H_21_N_3_O_3_	Insoluble
**4c**	Oxalate	62.0	151	0.50	C_17_H_23_N_3_O_7_	Highly soluble
**4d**	Citrate	90	148	0.13	C_21_H_2_9N_3_O_10_	Highly soluble

mp=Melting point

**Table 2:** Infrared (IR) spectra of O-*para*-toluoyl-β-(morpholin-1-yl) propioamidoxime (**4b**) and quaternary ammonium salts: hydrochloride (**4a**), oxalate (**4c**), citrate (**4d**), KBr tablet, cm^-1^.

Compound	Stretching and bending vibrations of bonds, cm^‒1^, KBr tablet								

	ν_C=0_	ν_C=N_	δ_N‒H_	ν_C=C_	ν_N‒0_	ν_C‒O_	ν_C‒N_	ν_N(+)‒H_(ν_Csp3‒H_)	ν_N(‒H)2_; ν_COO−H_ and ν_O−H_
**4a**	1738	1662	1648	1605	1115	1282	1096	2546, 2666 (2855, 2923)	3215, 3309, and 3445
**4b**	1731	1634	1612	1612	1113	1268	1097	(2825, 2869, 2928, 2968)	3132, 3364, and 3483
**4c**	1752	1658	1613	1613	1175	1263, 1280	1079	2522, 2612, 2684; (2830, 2903)	3202, 3324, and 3435
**4d**	1772	1653	1600	1600	1129	1232	1100	2640, 2730 (2894, 2962)	3121,3525

KBr=Potassium bromide

**Table 3:** ^1^H NMR spectra of O-*para*-toluoyl-β-(morpholin-1-yl) propioamidoxime (**4b**) and quaternary ammonium salts: hydrochloride (**4a**), oxalate (**4c**), and citrate (**4d**).

Compound	Chemical shifts, δ, ppm (J, Hz)*							

	O (CH_2_)_2_	N (CH2)2 (4b); N(+)(CH_2_)_2_ (4a, 4c, 4d)	α-CH_2_	β-CH_2_	NH_2_	N(+) H	*Para*-CH_3_	C_6_H_4_
**4a**	3.42t (8.1)	3.93 m	2.78t (8.4)	3.20 m (8.4)	6.86	11.61	2.37	7.31d (8.1); 8.01d (8.1)
**4b**	3.53 m	2.32 m	2.26t (7.0)	2.54t (7.0)	6.56	‒	2.28	7.25d (8.0); 7.96d (8.0)
**4c**	3.04 m	3.75 m	2.55t (7.0)	3.16t (7.0)	6.71	11.58	2.32	7.27d (8.0); 7.97d (8.0)
**4d**	2.58 m	2.64 m	2.38t (7.0)	2.80t (7.0)	6.68	12.05	2.32	7.25d (8.0); 7.96d (8.0)

* Proton signals of carboxylate anions: 9.38 ppm (HOOCCOO^−^) (**4c**); 2.54 (OH), 3.61 m (CH_2_)_2_, 8.83 br (COOH) (4d).

1 H NMR=Proton nuclear magnetic resonance

**Table 4:** *In vitro* anti-tubercular activity of the β-aminopropioamidoxime derivatives **3ac** , **4a**, **4c**, and **4d** against DS (H37Rv), MDR MTB, and *M. bovis* strains*.

Compound	3a	3b	3c	4a	4c	4d	Rifampicin
MBC DS MTB H37Rv, µg/mL	1	0.01	0.01	4**	0.1	0.1	1 (20)*
MBC MDR MTB, µg/mL	2	0.01	0.01	-	0.1	0.1	2
MBC *M. bovis*, µg/mL	1	1	1	0.1	0.1	0.1	1

* The MBC value of **4a** in the DS MTB H37Rv strain was 4 µg/mL; under these experimental conditions rifampicin MBC value was 20 µg/mL. MDR=Multidrug-resistant, MTB=*Mycobacterium tuberculosis*, MBC=Minimum bactericidal concentration, *M. bovis*=*Mycobacterium bovis*, DS=Drug-sensitive

## MATERIALS AND METHODS

### Ethical approval

This study only involved laboratory testing of mycobacteria and did not include studies on animals or humans; therefore, no prior ethical approval was required.

### Study period and location

The studies presented in the article were carried out in three research centers located in Almaty, Kazakhstan. Study on the synthesis of the 5-aryl-3-[β-(piperidin-1-yl)ethyl]-1,2,4-oxadiazoles (**3a–c**) and O-aroyl-β-(morpholin-1-yl)propioamidoximes (**4a–d**) have been carried out since the January to November 2002 for the first group of compounds and since the Januare to December 2018 for the second group of compounds in the Laboratory of Chemistry of Synthetic and Natural Drugs of JSC A.B. Bekturov Institute of Chemical Sciences (http://ihn.kz/). During the same time periods, biological screening for DS and MDR strains of *M. tuberculosis* was carried out for the corresponding groups of compounds in the National Reference Bacteriological Laboratory of the National Scientific Center of Phthisiopulmonology (https://www.nncf.kz/). Screening on *M. bovis* strains was performed in March to June 2024 at LLP Scientific and Production Center Biovet, Almaty, Kazakhstan (https://www.emis.com/php/company-profile/KZ/ Nauchno-Proizvodstvennyi_Tsentr_Biovet), which works in the field of scientific research in the veterinary sector.

### Synthesis of β-aminopropioamidoximes derivatives

This research mainly involved the synthesis of two structural groups of β-aminopropioamidoxime derivatives: **3a–c** and **4a–d**. In essence, the starting compounds are β-aminopropioamidoximes **1a** and **1b** [β-amino group: piperidin-1-yl (**1a**); morpholin-1-yl (**1b**)]. O-aroyl derivatives of amidoxime **2a–c** and **4a–d** were formed during the interaction of β-aminopropioamidoximes **1a** and **1b** with the substituted benzoic acid chlorides. 5-Aryl-3-[β-(piperidin-1-yl)ethyl]-1,2,4-oxadiazoles (**3a-c**) were obtained following dehydration of β-piperidine derivatives **2a-c**.

The target compounds **3a–c** and **4a–d** were purified through recrystallization and characterized by physicochemical (yield, %, melting point [mp], °C, and mobility indicator in thin-layer chromatography [*R*_f_]) and spectral data (infrared [IR] spectroscopy and proton nuclear magnetic resonance [1H NMR] spectroscopy).

The IR spectra of compounds **3a–3c** and **4a–d** were obtained using an FSM2201 spectrometer (Infraspek, St. Petersburg, Russia) with KBr tablets. 1H spectra were recorded on a Bruker Avance III 500 MHz NMR spectrometer (Bruker, BioSpin GmbH, Rheinstetten, Germany). The residual non-deuterated solvent deuterated dimethyl sulfoxide (2.50 ppm) was used as a reference. Elemental analysis was performed using a CE440 elemental analyzer (Exeter Analytical, Inc., Shanghai, China). Mps were determined in glass capillaries using a Stuart melting point apparatus SMP30 (Bibby Scientific Ltd., Stone, Staffordshire, UK). The completion of the reaction and the purity of the synthesized product were monitored using thin-layer chromatography (TLC) plates of the Sorbfil brand (Sorbpolymer, Krasnodar, Russia), coated with silica gel CTX-1A, grain size 5–17 μm, and containing the UV-254 indicator. The benzene–ethanol mixture (1:3) was used. Sigma-Aldridge reagents were used without purification. The solvents for synthesis, recrystallization, and TLC analysis (ethanol, dimethylformamide [DMF], isopropanol [i-PrOH], chloroform, benzene, acetone, and ethyl acetate [EtOAc]) were purified using known methods.

5-Aryl-3-[β-(piperidin-1-yl)ethyl]-1,2,4-oxadiazoles (**3a**-**c**) were obtained through O-aroyl-(2-piperidin-1-yl)propioamidoximes (**2a**-**c**) dehydration in DMF at 70°C (Scheme 2), as previously described by Kayukova *et al*. [44, 45] and β-(morpholin-1-yl)propioamidoxime **(1b)**. Morpholine (15.00 g; 0.172 mol) in 50 mL of absolute ethanol was placed in a 250 mL three-horned flask equipped with a refrigerator, stirrer, and drip funnel. A freshly distilled acrylonitrile (9.12 g; 0.172 mol) in 20 mL of absolute ethanol was added dropwise at mixing and cooling up to 15°C with cold water. The reaction mixture was mixed at room temperature (RT; 20°C–22°C) for 20 h; β-(morpholin-1-yl)propionitrile has a TLC stain at *R*_f_ 0.80.

Hydroxylamine hydrochloride (11.95 g; 0.172 mol) was added to the reaction mixture. Everything was mixed until a homogeneous state was achieved. The sodium ethylate (Na^+^C_2_H_5_O^-^) solution obtained from 3.95 g (0.172 mol) of metallic Na and 50 mL of absolute ethanol was added dropwise at 10°C while stirring. The reaction mixture was mixed at RT for 3 days; a precipitate, a mixture of β-(morpholin-1-yl)propioamidoxime (**1b**) and sodium chloride (NaCl), was filtered. Precipitation of **1b** gathered from evaporated alcohol filtrate and the extraction of a mixture of amidoxime **1b** and NaCl in the Soxhlet’s apparatus were crystallized from i-PrOH with β-(morpholin-1-yl)propioamidoxime (**1b**). This yields 9.65 g (66%), mp 136 °C, *R*_f_ 0.29. Anal. Calcd for C_7_H_15_N_3_O_2_ (173.12), %: C, 48.54; H, 8.73. Found: C, 48.06; H, 8.95.

Hydrochloride (**4a**). *para*-Toluoylchloride (0.32 g; 0.0021 mol) in 5 mL of dried EtOAc was added with stirring to 0.36 g (0.0021 mol) β-(morpholin-1-yl)propioamidoxime (**1b**) in 30 mL of dried EtOAc. The reaction mixture was stirred at RT for 6 h. The precipitate of technical **4a** was filtered and recrystallized from i-PrOH. Hydrochloride **4a** (0.32 g; 83.2%), mp 124 °C, *R*_f_ 0.46 was obtained. Anal. Calcd for C_15_H_22_ClN_3_O_3 (_327.81), %: C, 54.96; H, 6.76; Cl, 10.82. Found, %: 55.35; H, 6.95; Cl, 9.78.

O-*para*-Toluoyl-β-(morpholin-1-yl)propioamidoxime (**4b**). K_2_CO_3_ (0.25 g; 0.0018 mol) was added to 0.32 g (0.0018 moL) of O-*para*-toluoyl-β-(morpholin-1-yl)propioamidoxime hydrochloride (**4a**) in 10 mL of distilled water. Precipitate **4b** was formed after stirring at RT for 1 h; its recrystallization from i-PrOH yielded 24.18 g (93.0%) O-*para*-toluoyl-β-(morpholin-1-yl)propioamidoxime (**4b**) with mp 115°C, *R*_f_ 0.76. Anal. Calcd for C_15_H_21_N_3_O_3_ (291.35), %: C, 61.84; H, 7.27. Found, %: C, 62.20; H, 7.55.

Оxalate (**4c**). O-*para*-Toluoyl-β-(morpholin-1-yl)propioamidoxime (**4b**) (0.5 g; 0.0017 mol) and oxalic acid (0.15 g; 0.0017 mol) in 50 mL of acetone were refluxed under TLC control for 5 h. The precipitated salt was filtered, dried, and recrystallized from ethanol; oxalate **4c** yield was 0.4 g (62%), mp 140°C, *R*_f_ 0.60. Anal. Calcd for C_17_H_22_N_3_O_7_ (380.15), %: C, 53.68; H, 5.83. Found: C, 53.37; H, 5.95.

Citrate (**4d**). O-*para*-Toluoyl-β-(morpholin-1-yl)propioamidoxime (**4b**) (1 g; 0.0034 mol) and citric acid (0.65 g; 0.0034 mol) in 100 mL of acetone were stirred under TLC control for 30 min; a white matter precipitate was formed throughout the reaction mixture. The precipitate **4d** was filtered by adding 10 mL of acetone. Citrate **4d** (1.48 g; 90%), mp 54°C, *R*_f_ 0.75, was obtained following recrystallization from ethanol. Calcd for C_21_H_29_N_3_O_10_ (483.47), %: C, 52.17; H, 6.05. Found, %: C, 52.57; H, 6.38.

All compounds presented here are water-soluble, which is necessary for the oral administration of antitubercular drugs.

### *In vitro* bactericidal anti-tubercular activity and MBC in Sotton’s liquid medium

The compounds presented herein were selected based on the MBC values of < 100 μg/mL and the technological availability of samples from an array that had undergone *in vitro* screening against DS *M. tuberculosis* H37Rv and MDR *M. tuberculosis*. The selected samples were candidates for *in vitro* *M. bovis* screening.

#### The strains tested

DS *M. tuberculosis* H37Rv is a museum strain obtained from the National Scientific Center of Phthisiopulmonology strains collection, a wild strain of *M. tuberculosis* isolated from a patient in the National Scientific Center of Phthisiopulmonology clinic, typed as MDR, resistant to rifampicin and isoniazid; *M. bovis* is a museum strain received from the strain collection stored at JSC Kazakh Research Veterinary Institute.

#### Nutrient mediums

*In vitro* screening was performed on nutrient mediums: Sotton’s liquid medium (HiMedia M1276-500G Sauton’s Fluid Medium Base) and Lowenstein-Jensen dense medium prepared from the base (TB-Medium Base according to Lowenstein-Jensen, Sigma Aldrich, Catalog no. 63237) was obtained from JSCKelun-Kazpharm.

#### Standard used

Rifampicin (AppliChem, Darmstadt, Germany) was used as a standard for comparison with the tested drugs because it is commonly used in TB treatment.

The studied drug concentration ranged from 100 to 0.01 μg/mL based on 10-fold dilutions. For each drug concentration, three test tubes were used, and the experiment was conducted in two stages with two series. A mycobacterial culture (14–21 days) grown on a dense egg medium was removed from the slants under sterile conditions using a platinum spatula, ground in a test tube, and suspended in a 0.9% NaCl solution (physiological solution; TOOKelun-Kazpharm). After settling the large particles of the culture, the test tube was kept at RT for 20 min. Suspended particles of the generous size of the culture were allowed to settle, and the test tube was kept at RT for 20 min.

The bacterial suspension was collected using a pipette and transferred to another test tube. Muddiness, corresponding to the 5^th^ standard, was achieved by adding the physiological solution to the test tube; 1 mL of suspension corresponded to the 5^th^ standard of optical density containing 5 × 10^8^ microbial bodies.

A 0.1 mL suspension of *M. tuberculosis* in 0.2 mL of physiological solution was added to each test tube with different concentrations of the studied drugs. The control tube contained a nutrient medium without the drug. The tubes were incubated at 37°C for 10 days in Sotton’s liquid medium, following which the sediments were centrifuged and washed with physiological solution, and the washings were inoculated on the dense Lowenstein-Jensen medium. The growth of the colonies was recorded after 1 and 2.5 months [46].

Rifampicin was added to the *M. tuberculosis* solution at a concentration equal to that of the test drugs and incubated for the same incubation period as the test drugs in the other tubes.

The results of the cultural studies were recorded after 21–28 days and 2.5 months of cultivation in a thermostat at 37 °C according to the following scheme:

0: No MBT colonies on the shoal;

+: Single, up to 20 MBT colonies;

++: 20–100 MBT colonies;

+++: > 100 MBT colonies.

Table 4 lists the bactericidal concentrations of compounds **3a–c, 4a, 4c,** and **4d**, corresponding to values of 0 from the given designations.

### Software for *in silico* SwissADME prediction

ChemDraw Ultra Software V.12.0.2 and Spartan’14V 1.1.4 (http://www.swissadme.ch) [47] were used for in silico SwissADME prediction.

## RESULTS

### Physicochemical data and structure

1,2,4-Oxadiazoles (**3a**-**c**) were obtained by dehydration in DMF at 70°C of O-aroyl derivatives (**2a**-**c)** (Scheme 2) [44]. β-(Morpholin-1-yl)propioamidoxime (**1b**) was prepared using a one-pot method, the first stage of which was the reaction of morpholine with acrylonitrile in ethanol to form β-(morpholin-1-yl) propionitrile followed by the reaction with hydroxylamine in this same reactionary environment. Amidoxime **1b** was isolated with a yield of 66% [45].

Interaction of amidoxime (**1b**) and *para*-toluoyl chloride in EtOAc formed hydrochloride (**4a**) with a yield of 83.2%. O-*para*-toluoyl-β-morpholinopropioamidoxime oxalate (**4c**) was obtained by the interaction of equivalent amounts of O-*para*-toluoyl-β-morpholinopropioamidoxime (**4b**) and oxalic acid in boiling acetone with a yield of 62%. Using the same method, when O-*para*-toluoyl-β-morpholinopropioamidoxime (**4b**) was reacted with citric acid, citrate (**4d**) was formed with a yield of 90% (Table 1).

The IR spectra of compounds **4a**, **4c**, and **4d** showed evidence of ammonium compound formation: the stretching vibration double bands ν_C=O_ and ν_C=N_ shifted to higher wave numbers. In addition, in the Fourier transform IR spectra of the salts **4a**, **4c**, and **4d,** a set of stretching vibration bands of the ammonium bonds appeared in the region 2522-2730 cm^-1^ (Table 2).

As a rule, in the 1H NMR spectra, the formation of a quaternary charged nitrogen atom in **4a**,**4c**, and **4d** shifts the proton signals of the α- and β-methylene groups and the NH_2_ groups’ proton signals to lower fields. In 1H NMR spectra of **4c** and **4d**, the signals of the carboxylate anions protons appeared (Table 3).

In 1H NMR spectra of **4c** and **4d**, the signals of the carboxylate anions protons appeared.

### *In vitro* antitubercular screening of the β-aminopropioamidoximes derivatives 3a-c, 4a, 4c, and 4d against *M. tuberculosis* and *M. bovis*

As shown by *in vitro* screening (Table 4), compounds **3ac, 4a, 4c**, and **4d** demonstrated significant bactericidal activity, inhibiting the growth of DS and MDR *M. tuberculosis* and *M. bovis* strains. The MBC of rifampicin against *M. tuberculosis* H37Rv strain was 20 and 1 μg/mL, against the MDR wild strain of *M. tuberculosis* was 2 mkg/mL, and against *M. bovis* was 1 μg/mL.

The comparison of the MBC values of the β-aminopropioamidoxime derivatives **3a**-**c**, **4a**, **4c**, and **4d** with the MBC values of rifampicin showed that the compounds under study have the following properties:

- 1,2,4-Oxadiazoles **3a-c** have MBC values equal to rifampicin - 1 μg/mL against *M. bovis* and 2 μg/mL against MDR *M. tuberculosis* (**3a**).
- O-*para*-Toluoyl-β-morpholinopropioamidoxime salts: hydrochloride (**4a**), oxalate (**4c**) and citrate (**4d**) have MBC values 5–10 times lower (4 and 0.1 μg/mL) than that of rifampicin (20 and 1.0 μg/mL) against DS M. tuberculosis H37Rv strain. Oxalate (**4c**) and citrate (**4d**) show MBC (0.1 μg/mL) against MDR *M. tuberculosis* strain, Что 20 times lower than rifampicin (2 μg/mL). Also, the entire set of salts (**4a**, **4c**, and **4d**) had a 10-fold lower MBC (0.1 μg/mL ) against *M. bovis* strain than rifampicin (1 μg/mL).
- 1,2,4-Oxadiazoles **3b** and **3c** against the DS strain of *M. tuberculosis H37Rv* show MBC values 100 times lower than those of rifampicin MBC (0.01 μg/mL and 1 μg/mL, respectively).
- At the same time, these compounds **3b** and **3c** against MDR *M. tuberculosis* strain show MBC values 200 times lower than those of rifampicin (0.01 μg/mL and 2 μg/mL, respectively).

However, when switching from *in vitro* screening of 1,2,4-oxadiazoles **3b** and **3c** against *M. tuberculosis* to *in vitro* screening against *M. bovis*, there is no parallelism in maintaining the bactericidal effect. Their MBC against *M. bovis* decreased from 0.01 to 1 μg/mL.

### *In silico* absorption, distribution, metabolism, and excretion (ADME)/pharmacokinetic predictions

**Figure 3:**
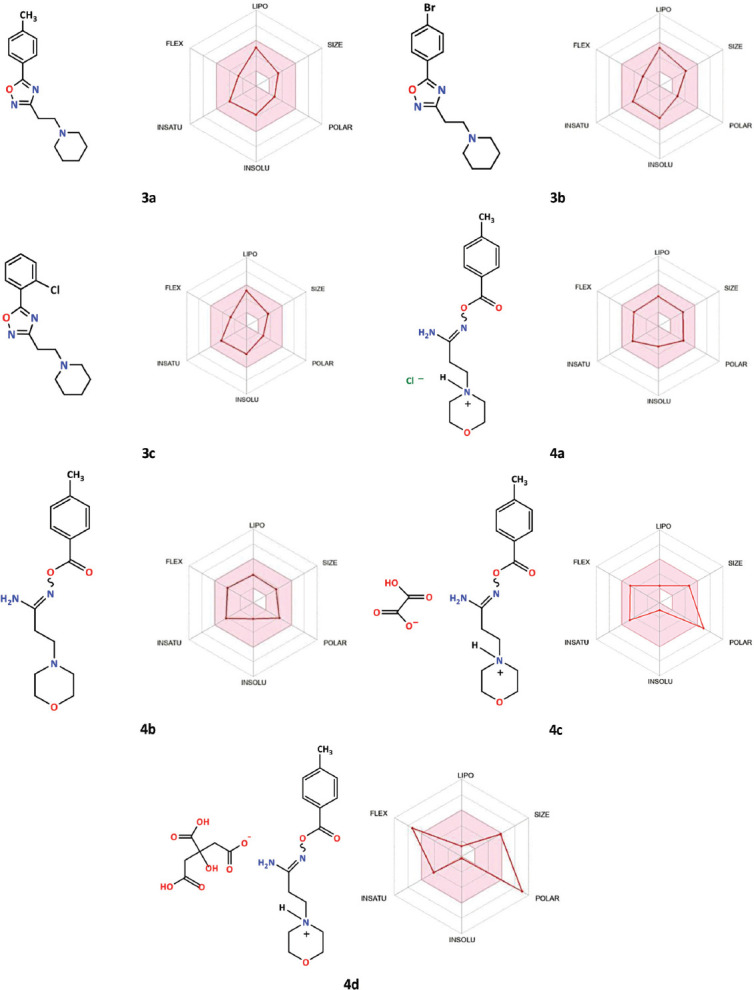
Structure and bioavailability radars of the β-aminopropioamidoxime derivatives **3a-b** and **4a-d**.

Based on bioavailability radar, initial assessment displayed varying degrees of drug-likeness of β-aminopropioamidoximes derivatives **3a-c** and **4a-d** (Figure 3).

The graphical images of the molecular radars and ADME drug-likeness parameters were obtained based on their molecular input line SMILES (simplified molecular input line entry system) (Tables 5 and 6), which were formed after entering into the SwissADME system the structures of molecules depicted in the ChemDraw program.

**Table 5:** SMILES for calculating ADME *para*meters.

Compound	SMILES
**3a**	CC1=CC=C (C=C1) C1=NC (CCN2CCCCC2)=NO1
**3b**	BrC1=CC=C (C=C1) C1=NC (CCN2CCCCC2)=NO1
**3c**	ClC1=CC=CC(=C1) C1=NC (CCN2CCCCC2)=NO1
**4a**	[Cl-].[H][N+]1(CC\C (N)=N\OC(=O) C2=CC=C (C) C=C2) CCOCC1
**4b**	CC1=CC=C (C=C1) C(=O) O\N=C(/N) CCN1CCOCC1
**4c**	OC(=O) C([O-])=O.[H][N+]1(CC\C (N)=N\OC(=O) C2=CC=C (C) C=C2) CCOCC1
**4d**	OC(=O) CC (O)(CC([O-])=O) C (O)=O.[H][N+]1(CC\C (N)=N\OC(=O) C2=CC=C (C) C=C2) CCOCC1

SMILES=Simplified molecular input line entry system, ADME=Absorption, distribution, metabolism, and excretion

**Table 6:** ADME and drug-likeness *para*meters of β-aminopropioamidoxime derivatives.

Indicator	3a	3b	3c	4a	4b	4c	4d
Physicochemical properties							
MW (g/mol)	271.36	336.23	291.78	327.61	291.35	381.36	483.47
nHBA	4	4	4	4	5	8	11
nHBD	0	0	0	2	1	3	5
TPSA(Å)	42.16	42.16	42.16	78.356	77.15	155.76	213.31
Lipophilicity							
iLOGP	3.70	3.81	3.71	0.00	2.62	2.26	1.93
Water solubility							
Class	Soluble	Moderately soluble	Moderately soluble	Soluble	Soluble	Very soluble	Very soluble
Pharmacokinetics							
GI absorption	High	High	High	High	High	High	High
BBB permeant	Yes	Yes	Yes	No	No	No	No
Drug-likeness							
Lipinski	Yes; 0 V	Yes; 0 V	Yes; 0 V	Yes; 0 V	Yes; 0 V	Yes; 0 V	Yes; 1 V N or O >10
Bioavailability Score	0.55	0.55	0.55	0.55	0.55	0.55	0.11
Medicinal chemistry							
Lead likeness	Yes	No; 1V: XLOGP3 >3.5	No; 1: XLOGP3 >3.5	Yes	Yes	Yes	No; 1V: Rotors >7
Synthetic accessibility	3.07	2.99	2.97	3.10	3.03	3.35	4.36

V=Violation, MW=(g/mol) molecular weight, nHBA=Number of hydrogen bond acceptor, nHBD=Number of hydrogen bond donor, TPSA=Topological polar surface area, iLOGP-Octanol/water partition coefficient, SA=Synthetic accessibility, GI=Absorption gastrointestinal absorption, BBB=Permeant blood–brain barrier permeant, ADME=Absorption, distribution, metabolism, and excretion

Six physicochemical properties were considered: lipophilicity, size, polarity, solubility, flexibility, and saturation, displayed on each axis using optimal descriptor values [47].

A molecule is recognized as drug-like if its Radar plot falls within the pink area [48]. When examining the Bioavailability Radar plots of compounds **3a**-**3c** and **4a-4d**, we see that the first 5 compounds **3a**-**3c**, **4a**, and **4b** fall within the pink hexagon; to a first approximation, this allows us to consider them drug-like. The Radar plots of compounds **4c** and **4d** indicate their lower bioavailability due to the polarity of compound **4c** and the polarity and flexibility of compound **4d**.

ADME parameters can help to make informed decisions on the selection of promising drug-like compounds from the beta-aminopropioamidoxime derivatives studied at the *in vitro* stage. These parameters evaluate drug-like candidates that can be introduced into a biological system, taking into account their similarity to drugs (Table 6) [49, 50].

To assess drug similarity and determine whether the test compound will be orally active in humans, the Lipinski rule of five (Ro5) [51] is proposed. It is assumed that a molecule or an inhibitor can be orally absorbed/active if two (2) or more of these starting points satisfy the following conditions: Molecular weight (MW) of molecule <500, octanol/water partition coefficient (iLOGP) ≤ 5, number of hydrogen bond acceptors (nHBA) ≤ 10, and number of hydrogen bond donors (nHBD) ≤ 5.

From the meanings of drug-likeness properties shown in Table 5, it was observed that **3a–3c** and **4a–4d** molecules have zero violations of Lipinski’s rule. Thus, the MWs of all compounds <500 g/mol), iLOGP is in the range of 0.00 ÷ 3.81; the number nHBA ≤ 10 except for compound **4d**; and the numbers nHBD are in the range of 0 ÷ 5. Thus, based on the Physicochemical Properties and Lipophilicity given in Table 5, it can be concluded that the compounds under study are drug-like.

Drug-likeness parameters are related to aqueous solubility, and intestinal permeability determines the first step of oral bioavailability [47].

Topological polar surface area (TPSA) values reflect the area of the polar molecule and are increased as the number of polar groups in the molecule increases; functional groups containing nitrogen and oxygen atoms contribute to polarity and increase the TPSA value for a drug [52]. This feature is observed in the raw data of compounds **3a**–**3c** and **4a–4d**; the TPSA values vary from 42.16 to 213.31 Å^2^.

Drugs with higher TPSA values are less lipid-soluble and will, in general, be absorbed less extensively and more slowly and will distribute less extensively than drugs with lower TPSA values. TPSA values are related to gastrointestinal absorption and blood-brain barrier (BBB) crossing. All compounds have a high gastrointestinal absorption (HI) value ‒ “High.” Different “blood–Brain Barrier Permeant” values for a series of 1,2,4-oxadiazoles (**3a**-**c**) ‒ “High” and for a series of **4a–d** - “No” demonstrates how increasing polarity in the second row reduces the ability to penetrate the BBB.

Bioavailability defines the ability of a drug molecule to access cells by crossing cell membranes. A bioavailability score, BS, is described as the probability that a compound will have >10% bioavailability in rats. It defines four classes of compounds with probabilities to cross cell membranes of 11%, 17%, 56%, or 85%, the TPSA values of which fall into 4 different areas: TPSA is >150 Å^2^ (11%); TPSA is between 75 and 150 Å^2^ (56%); and TPSA is <75 Å^2^ (85%). The remaining compounds have a BS equal to 0.55 if they pass the rule-of-five and 0.17 if they fail [53].

The BS of compounds **3a**-**c** and **4a-d** are represented by two values: 0.55 for compounds **3a–c** and **4a–c** and 0.11 for compound **4d**. The latter compound had the largest value of TPSA 213.31 Å^2^ and one deviation from Lipinski’s rule, while the TPSA area of the first group of compounds is 42.16 ÷ 155.76 Å^2^. These data are in approximate agreement with the aforementioned limitations [53], and the most polar compound should have the lowest bioavailability.

In the studied series, lead-likeness was established for four out of seven compounds; this indicator is inherent in 5-toluene-3-[β-(piperidin-1-yl)ethyl]-1,2,4-oxadiazole and O-toluoyl-β-(morpholin-1-yl)propioamidoxime hydrochloride, base, and oxalate.

Derivatives of β-aminopropioamidoximes have acceptable synthetic availability in the range of 2.97÷4.36, which is closer to the easy level under the condition «1» ‒ easily available and «10» ‒ difficult to access.

## DISCUSSION

According to administrative data of the healthcare system of the Republic of Kazakhstan on the epidemiology and mortality of tuberculosis in Kazakhstan, of the 149,122 patients with TB, from 2014 to 2019, TB incidence declined from 227 to 15.2 per 100,000 individuals, while all-cause mortality increased from 8.4 to 15.2 per 100,000 individuals [54]. The following comorbid conditions that complicate treatment, such as HIV [55], DM [56], stroke [57], chronic kidney disease (CKD) [58], and liver cirrhosis [59] were present in patients with TB.

Despite WHO recommendations to treat TB in an outpatient setting, Kazakhstan still has a high hospitalization rate at TB centers [60]. The latter circumstance led to an increased risk of in-hospital transmission of drug-resistant TB and is the decisive reason of the mortality rate during 2014–2019.

In Kazakhstan, the prevalence of DR-TB remains on average at the level of 45%–49% of all registered TB cases, which was the basis for its inclusion in the list of countries in Eastern Europe and Central Asia with a high burden of DR-TB [61]. Kazakhstan recognizes the importance of achieving the goal of “Ending the TB Epidemic by 2030” set out in the Sustainable Development Goals, the Political Declaration to Combat Tuberculosis, endorsed by the UN General Assembly, and the World Health Organization’s Stop TB Strategy [62].

Treatment of patients with active tuberculosis in Kazakhstan is carried out with anti-tuberculosis drugs within the framework of the guaranteed volume of free medical care. Within the framework of the implementation of pilot projects, all-oral injection-free, shortened treatment regimens for MDR TB with new anti-TB drugs are used:

1) Levofloxacin (Lfx), bedaquiline (Bdq), linezolid (Lzd), clofazimine (Cfz), and delamanid (Dlm). 2) Bedaquiline (Bdq), linezolid (Lzd), levofloxacin (Lfx), clofazimine (Cfz), and cycloserine (Cs) [pyrazinamide (Z)]. The total course of treatment is 6–9 months with daily administration of drugs. The duration of use of bedaquiline and delamanid is 6 months. Clinically-relevant adverse events of special interest were uncommon. All regimens demonstrated excellent safety and effectiveness, expanding the potential treatment options for patients, providers, and programs [63, 64].

The problem of Kazakhstan’s pharmaceutical science, which is probably a problem for many middle-income countries, is that the local pharmaceutical industry produces mainly generics, while original products are imported from abroad. However, new pharmaceuticals are being developed by scientists in Kazakhstan, regardless of the difficult and lengthy path of introduction into medical practice. An example of this are our works [41–43].

In addition, within the framework of the Development of New Anti-Infective Drugs program (JSC Scientific Center for Anti-Infective Drugs, Almaty, Kazakhstan) for many years, it was created as an original anti-tuberculosis drug FS-1 which is an ionic nanostructured complex of carbohydrates of proteins and polypeptides, iodine, and halides of alkaline and alkaline earth elements. FS-1 is highly effective against various Gram-positive and Gram-negative bacteria, including those resistant to antibiotics. MIC FS-1 varied over a wide range from 0.02 to 0.3 mg/mL. The main mechanism of action of FS-1 consists in membranolytic activity [65]. At present, this drug has passed the phase 3 of clinical trials [66].

Aligned with the “One Health” approach [22-24], this study identified a series of β-amino-propioamidoxime derivatives as highly active, water-soluble, and technologically advanced candidates for antitubercular therapy. These compounds demonstrated remarkable activity, showing 5–10 times higher efficacy against human strains of *M. tuberculosis* H37Rv, 20 times greater effectiveness against MDR *M. tuberculosis* strains, and a 10-fold improvement against zoonotic infections caused by *M. bovis*.

A comparative analysis of MBCs, synthetic pathways, and physicochemical properties of the β-aminopropioamidoximes derivatives with current drugs in the short oral regimen for MDR TB (including bedaquiline, pretomanid, linezolid, moxifloxacin, and clofazimine) highlighted the distinct advantages of these new derivatives. The notable *in vitro* activity, water solubility, simplified synthesis process, and overcoming drug resistance, along with the significant adverse effects associated with existing anti-tuberculosis drugs, underscores the potential of β-aminopropioamidoximes derivatives as a promising new class of anti-TB agents.

The simultaneous screening of β-aminopropio-amidoximes derivatives against DS and MDR human *M. tuberculosis*, as well as zoonotic *M. bovis* strains, offers a holistic strategy for addressing critical health challenges and advancing the eradication of TB infections through new, highly potent therapeutic options.

The anti-tubercular screening results revealed that all tested β-aminopropioamidoximes derivatives, except compound **3a**, demonstrated MBC values 5–100 times lower than rifampicin against DS and MDR strains of *M. tuberculosis*. Notably, compound **3a** exhibited activity comparable to rifampicin. Against *M. bovis*, compounds **3a**–**c** showed equivalent activity to rifampicin, whereas salts **4a**, **4c**, and **4d** demonstrated tenfold higher efficacy against all tested strains. Compounds **3b** and **3c**, being 100 times more potent than rifampicin, exhibit promising potential for clinical applications in treating DS and MDR human TB. The introduction of new active molecules for managing anthropozoonotic infections caused by human DS and MDR *M. tuberculosis* and *M. bovis* strains is therefore requested.

*In silico* ADME analysis affirmed the strong drug-likeness of β-aminopropioamidoxime derivatives, with favorable pharmacokinetic characteristics, including high solubility and efficient gastrointestinal absorption. The analysis indicated the ability of 1,2,4-oxadiazoles to permeate the BBB and the lack of this property in certain salts and the base of O-toluoyl-β-(morpholin-1-yl)propioamidoxime.

The bioavailability values revealed the varied capacity of β-aminopropioamidoxime derivatives to traverse cell membranes, with O-toluoyl-β-(morpholin-1-yl)propioamidoxime citrate showing the lowest bioavailability due to its high polarity. Among the tested compounds, four demonstrated lead-likeness, and the derivatives of β-aminopropioamidoximes showed acceptable synthetic accessibility.

Considering the high costs and prolonged duration of current MDR TB treatments, along with the simplified synthesis of β-aminopropioamidoximes derivatives achieving quantitative yields at every stage, this study emphasizes the potential of these compounds as a novel group of anti-tubercular agents. However, a major limitation in developing new anti-tubercular drugs is the financial and temporal burden of extensive preclinical and clinical trials, which is particularly challenging for countries with constrained research budgets, such as Kazakhstan.

## CONCLUSION

This study introduces a novel library of β-aminopropioamidoxime derivatives with promising antitubercular properties against both human and zoonotic TB (zTB) strains. The synthesized compounds exhibited remarkable *in vitro* efficacy, demonstrating 5–100 times greater activity than rifampicin against DS and MDR *M. tuberculosis* and *M. bovis* strains. Notably, 1,2,4-oxadiazoles with *para*-bromo and *meta*-chloro substituents on the phenyl ring exhibited the highest potency against *M. tuberculosis*, while O-*para*-toluoyl-β-(morpholin-1-yl)propioamidoxime salts showed significant activity against *M. bovis*. The *in silico* ADME analysis supported the drug-likeness of these compounds, highlighting their favorable pharmacokinetic profiles, including good solubility, high gastrointestinal absorption, and selective BBB permeability.

The study successfully integrated a “One Health” approach by evaluating the compounds against both human and zTB strains, enhancing the potential for broad-spectrum therapeutic applications. The combined *in vitro* and *in silico* analyses provided a robust assessment of the compounds’ efficacy, bioavailability, and drug-likeness, offering a strong foundation for further development. In addition, the compounds’ simple synthetic routes, high yields, and water solubility align well with the requirements for practical drug development.

Despite the promising *in vitro* results, the study is limited by the absence of *in vivo* evaluations, which are critical to establishing the therapeutic efficacy and safety of β-aminopropioamidoxime derivatives in living organisms. In addition, potential toxicity and long-term safety profiles were not assessed, necessitating comprehensive preclinical studies. The study’s findings are also confined to laboratory conditions, which may not fully replicate clinical settings.

Future research should focus on *in vivo* studies to validate the antitubercular efficacy of β-aminopropioamidoxime derivatives in animal models. Pharmacokinetic and toxicological assessments are essential to ensure safety and therapeutic viability. The compounds showing selective BBB permeability could also be explored for treating central nervous system TB infections. In addition, optimization of lead compounds and development of formulations suitable for oral administration could facilitate their integration into treatment regimens for both human and animal TB, contributing to the global effort to eradicate TB in alignment with the “One Health” framework.

Overall, this study presents β-amino-propioamidoxime derivatives as promising candidates for new antitubercular therapies, with the potential to address the challenges of MDR TB and zTB infections effectively.

## AUTHORS’ CONTRIBUTIONS

LK, UJ, VB, KT, and LC: Conceptualization; LK, AU, UJ, VB, KT, and LC: Methodology and investigations; AU, MM, GD, GB, and AY: Chemical experiment and its description; VB, LC, and AA: Microbiological screening for *M. tuberculosis* and its description; KT and AB: Microbiological screening for *M. bovis* and its description; LK and AY: Visualizations and SwissADME. LK: Drafted and revised the manuscript. All authors have read and approved the final manuscript.

## References

[1] WHO (2023). Global Tuberculosis Report 2023. Geneva:World Health Organization 2023.

[2] Cilloni L, Fu H, Vesga J.F, Dowdy D, Pretorius C, Ahmedov S, Nair A, Mosneaga S, Masini A, Sahu S, Arinaminpathy N (2020). The potential impact of the COVID-19 pandemic on the Tuberculosis epidemic a modelling analysis. EClinicalMedicine.

[3] Kayukova L.A, Berikova E.A (2020). Modern anti-TB drugs and their classification. Part I. First-line drugs (review). Pharm. Chem. J.

[4] Maitre T, Baulard A, Aubry A, Veziris N (2024). Optimizing the use of current antituberculosis drugs to overcome drug resistance in *Mycobacterium tuberculosis*. Infect. Dis. Now.

[5] Pontali E, Raviglione M.C, Migliori G.B, The Writing Group Members of the Global TB Network Clinical Trials Committee (2019). Regimens to treat multidrug-resistant tuberculosis:past, present and future perspectives. Eur. Respir. Rev.

[6] United Nations (2023). Political Declaration of the High-level Meeting of the General Assembly on the Fight against Tuberculosis. United Nations, New York.

[7] Akalu T, Clements A, Wolde H, Alene K.A (2023). Economic burden of multidrug-resistant tuberculosis on patients and households:A global systematic review and meta-analysis. Sci. Rep.

[8] WHO Launches the TB Research Tracker an Online Platform to Track Progress in TB Research.

[9] WHO (2022). WHO Consolidated Guidelines on Tuberculosis. Module 4:Treatment - Drug-resistant Tuberculosis Treatment, 2022 Update. World Health Organization, Geneva.

[10] Barbier M, Wirth T (2016). The Evolutionary history, demography, and spread of the *Mycobacterium tuberculosis* complex. Microbiol Spectr.

[11] Brites D, Loiseau C, Menardo F, Borrell S, Boniotti M.B, Warren R, Dippenaar A, Parsons S.D.C, Beisel C, Behr M.A, Fyfe J.A, Coscolla M, Gagneux S (2018). A new phylogenetic framework for the animal adapted mycobacterium tuberculosis complex. Front. Microbiol.

[12] Orgeur M, Brosch R (2018). Evolution of virulence in the *Mycobacterium tuberculosis* complex. Curr. Opin. Microbiol.

[13] Otto-Knapp R, Schenkel K, Bauer T (2016). Standard therapy of tuberculosis. Internist (Berl.).

[14] Lan Z, Bastos M, Menzies D (2016). Treatment of human disease due to *Mycobacterium bovis*:A systematic review. Eur. Respir. J.

[15] World Health Organization (2018). Global Tuberculosis Report 2018.

[16] WHO (2019). WHO Global Tuberculosis Report.

[17] El-Sayed A, El-Shannat S, Kamel M, Castaneda-Vazquez M.A, Castaneda-Vazquez H (2016). Molecular epidemiology of *Mycobacterium bovis* in humans and cattle. Zoonoses Public Health.

[18] WOAH Terrestrial Manual 2022 Chapter 3.1.1 3 Mammalian Tuberculosis (Infection with mycobacterium tuberculosis complex).

[19] Turgenbayev K.A, Borsynbayeva A.M, Plazun A.A, Turgenbayev R.K (2021). Tuberculosis prevalence in animals and humans in the Republic of Kazakhstan. Vet. World.

[20] Zinsstag J, Schelling E, Roth F, Kazwala R.R (2008). Economics of Bovine Tuberculosis:*Mycobacterium bovis* Infection in Animals and Humans. Iowa State University Press, Ames.

[21] WHO https://www.who.int/news-room/questions-and-answers/item/tuberculosis-multidrug-resistant-tuberculosis-(mdr-tb).

[22] Walther B, Schaufle K, Wieler L.H, Lübke-Becker A, Sing A (2022). Zoonotic and multidrug-resistant bacteria in companion animals challenge infection medicine and biosecurity. Zoonoses:Infections Affecting Humans and Animals. Springer, Cham.

[23] One Health Initiative http://www.onehealthinitiative.com.

[24] de Macedo Couto R, Santana G.O, Ranzani O.T, Waldman E.A (2022). One Health and surveillance of zoonotic tuberculosis in selected low-income, middle-income and high-income countries:A systematic review. PLoS Negl. Trop. Dis.

[25] Berry C, du Cros P, Fielding K, Gajewski S, Kazounis E, McHugh T.D, Merle C, Motta I, Moore D.A.J, Nyang'wa B.T (2022). TB-PRACTECAL:Study protocol for a randomised, controlled, open-label, phase II-III trial to evaluate the safety and efficacy of regimens containing bedaquiline and pretomanid for the treatment of adult patients with pulmonary multidrug-resistant tuberculosis. Trials.

[26] Koul A, Vranckx L, Dendouga N, Balemans W, Van den Wyngaert I, Vergauwen K, Göhlmann H.W.H, Willebrords R, Poncelet A, Guillemont J, Bald D, Andries K (2008). Diarylquinolines are bactericidal for dormant mycobacteria as a result of disturbed ATP homeostasis. J. Biol. Chem.

[27] Zheng H, He W, Jiao W, Xia H, Sun L, Wang S, Xiao J, Ou X, Zhao Y, Shen A (2021). Molecular characterization of multidrug-resistant tuberculosis against levofloxacin, moxifloxacin, bedaquiline, linezolid, clofazimine, and delamanid in southwest of China. BMC Infect. Dis.

[28] Nuermberger E, Tyagi S, Tasneen R, Williams K.N, Almeida D, Rosenthal I, Grosset J.H (2008). Powerful bactericidal and sterilizing activity of a regimen containing pa-824, moxifloxacin, and pyrazinamide in a murine model of tuberculosis. Antimicrob. Agents Chemother.

[29] Yang C, Lei H, Wang D, Meng X, He J, Tong A, Zhu L, Jiang Y, Dong M (2012). *In vitro* activity of linezolid against clinical isolates of *Mycobacterium tuberculosis*, including multidrug-resistant and extensively drug-resistant strains from Beijing, China. Jpn J. Infect. Dis.

[30] Somasundaram S, Paramasivan N.C (2006). Susceptibility of *Mycobacterium tuberculosis* strains to gatifloxacin and moxifloxacin by different methods. Chemotherapy.

[31] Reddy V.M, O'Sullivan J.F, Gangadharam P.R (1999). Antimycobacterial activities of riminophenazines. J. Antimicrob. Chemother.

[32] McNevin M, Higgins J, Templeton A, Byrn S, Haskell R, Prisinzano T (2015). Strategies and methods for drug candidate phase optimization in discovery space. Discovering and Developing Molecules with Optimal Drug-like Properties. Vol. 15. Springer, New York.

[33] Okezue M, Bogdanowich-Knipp S, Smith D, Zeller M, Byrn S, Smith P, Purcell D.K, Clase K (2021). Salts and polymorph screens for bedaquiline. AAPSPharm. Sci. Tech.

[34] Dailymed - Linezolid - Linezolid Granule for Suspension (2022). Camber Pharmaceuticals Inc.

[35] Moxifloxacin Injection - Product Monograph (2020). Moxifloxacin Injection 400 mg/250 mL (1.6 mg/mL) (as Moxifloxacin Hydrochloride) Antibacterial Agent. Fresenius Kabi Canada Ltd., Toronto, ON.

[36] Stadler J.A.M, Maartens G, Meintjes G, Wasserman S (2023). Clofazimine for the treatment of tuberculosis. Front. Pharmacol.

[37] Lenaerts A.J, Gruppo V, Marietta K.S, Johnson C.M, Driscoll D.K, Tompkins N.M, Rose J.D, Reynolds R.C, Orme I.M (2005). Preclinical testing of the nitroimidazopyran PA-824 for activity against *Mycobacterium tuberculosis* in a series of *in vitro* and *in vivo* models. Antimicrob. Agents Chemother.

[38] (2022). New Tuberculosis Treatments (Drugs and Regimens).

[39] Bhagwat A, Deshpande A, Parish T (2022). *Mycobacterium tuberculosis* drug resistance has shaped anti-tubercular drug discovery. Front. Cell. Infect. Microbiol.

[40] World Health Organization (2020). Global Tuberculosis Report. WHO, Geneva.

[41] Kayukova L.A, Orazbaeva M.A, Bismilda V.L, Chingisova L.T (2010). Synthesis and antituberculosis activity of O-aroyl- -(4-phenylpiperazin-1-yl)propioamidoximes. Pharm. Chem. J.

[42] Kayukova L, Jussipbekov U, Praliyev K (2020). Amidoxime Derivatives with Local Anesthetic, Antitubercular, and Antidiabetic Activity. IntechOpen, London.

[43] Uzakova A.B, Kayukova L.A, Poroikov B.B, Praliev R.D (2018). A Comparison of the data of bioinformatics and experimental *in vitro* antitubercular activity of the new -aminopropioamidoximes library. Res. J. Pharm. Tech.

[44] Kayukova L.A, Zhumadildaeva I.S, Praliyev K.D (2002). Cyclization of O-benzoyl- -piperidinopropionamidoximes to form 5-phenyl-3-( -piperidino)ethyl-1,2,4-oxadiazoles. Russ. Chem. Bull.

[45] Kayukova L.A, Myrzabek A.B, Baitursynova G.P, Ergaliyeva E.M, Kurmangaliyeva A.B (2022). JSC Institute of Chemical Sciences. A Method for Producing of -(morpholin-1-yl) Propioamidoxime, an Intermediate Product in the Synthesis of a Substance with Anti-tubercular and Anti-diabetic activity. Kazakhstan Patent for Utility Model No. 6887.

[46] Golyshevskaya V.I, Guskova T.A, Shulgina M.V, Martynova L.P, Mozhokina G.N, Sokolova G.B (2012). Chapter 34:Methodological recommendations for studying the anti-tuberculosis activity of drugs. In:Korobov, N.V., (ed) The Digest of Articles:Guidelines for Conducting of Preclinical Studies of Drugs. Part 1., Vol. 1. Publication of the Federal State Budgetary Institution Scientific Center for Expertise of Medical Products of the Ministry of Health of the Russian Federation, Moscow.

[47] Daina A, Michielin O, Zoete V (2017). SwissADME:A free web tool to evaluate pharmacokinetics, drug-likeness and medicinal chemistry friendliness of small molecules. Sci. Rep.

[48] Ritchie T.J, Ertl P, Lewis R (2011). The graphical representation of ADME-related molecule properties for medicinal chemists. Drug Discov. Today.

[49] Attique S.A, Hassan M, Usman M, Atif R.M, Mahboob S, Al-Ghanim K.A, Bilal M, Nawaz M.Z (2019). A Molecular docking approach to evaluate the pharmacological properties of natural and synthetic treatment candidates for use against hypertension. Int. J. Environ. Res. Public Health.

[50] Bello A, Adamu U, Gideon U, Shallangwa A, Uba S (2020). Design of potential anti-melanoma agents against SK-MEL-5 cell line using QSAR modeling and molecular docking methods. SN Appl. Sci.

[51] Lipinski C.A, Lombardo F, Dominy B.W, Feeney P.J (1997). Experimental and computational approaches to estimate solubility and permeability in drug discovery and development settings. Adv. Drug Deliv. Rev.

[52] Ertl P, Rohde B, Selzer P (2000). Fast calculation of molecular polar surface area as a sum of fragment-based contributions and its application to the prediction of drug transport properties. J. Med. Chem.

[53] Martin Y.C (2005). A Bioavailability score. J. Med. Chem.

[54] Sakko Y, Madikenova M, Kim A, Syssoyev D, Mussina K, Gusmanov A, Zhakhina G, Yerdessov S, Semenova Y, Crape B.L, Sarria-Santamera A, Gaipov A (2023). Epidemiology of tuberculosis in Kazakhstan:data from the Unified National Electronic Healthcare System 2014–2019. BMJ Open.

[55] Shaweno D Worku A (2012). Tuberculosis treatment survival of HIV positive TB patients on directly observed treatment short-course in Southern Ethiopia:a retrospective cohort study. BMC Res. Notes.

[56] Ye Z, Li L, Yang L, Zhuang L, Aspatwar A, Wang L, Gong W (2024). Impact of diabetes mellitus on tuberculosis prevention, diagnosis, and treatment from an immunologic perspective. Exploration (Beijing).

[57] Zhang L, Zhang X, Li H, Chen G, Zhu M (2019). Acute ischemic stroke in young adults with tuberculous meningitis. BMC Infect. Dis.

[58] Xiao J, Ge J, Zhang D, Lin X, Wang X, Peng L, Chen L (2022). Clinical characteristics and outcomes in chronic kidney disease patients with tuberculosis in China:A retrospective cohort study. Int. J. Gen. Med.

[59] Jaisi B (2018). Prevalence of tuberculosis in patients with liver cirrhosis. J. Nepal Health Res. Counc.

[60] Darisheva M, Tracy M, Terlikbayeva A, Zhussupov B, Schluger N, McCrimmon T (2020). Knowledge and attitudes towards ambulatory treatment of tuberculоsis in Kazakhstan. BMC Health Serv. Res.

[61] https://www.who.int/news-room/fact-sheets/detail/tuberculosis.

[62] World Health Organization. The Moscow Declaration. First WHO Global Ministerial Conference Ending Tuberculosis in the Sustainable Development Era:A Multisectoral Response. Moscow Russian Federation, 16–17 November 2017.

[63] Ryskulov G.P, Adenov M, Turdaliyeva B, Maryandyshev A.O (2023). Short term regimens for drug-resistant tuberculosis:A review of clinical effectiveness and cost-effectiveness. Phthisiopulmonology.

[64] Keertan D, Christoph L (2024). Towards shorter, safer, flexible, and more effective treatment regimens for drug-resistant tuberculosis. Lancet Respir. Med.

[65] Islamov R, Kerimzhanova B, Ilin A (2019). New Antituberculosis Drug FS-1. Medicinal Chemistry. IntechOpen.

[66] FS-1 Drug for Treatment of Multiple Drug-resistant Tuberculosis (FS-1) ClinicalTrials.gov ID NCT02607449.

